# Necrosulfonamide Alleviates Acute Brain Injury of Intracerebral Hemorrhage *via* Inhibiting Inflammation and Necroptosis

**DOI:** 10.3389/fnmol.2022.916249

**Published:** 2022-06-02

**Authors:** Xiangyu Zhang, Yan Zhang, Fei Wang, Yang Liu, V. Wee Yong, Mengzhou Xue

**Affiliations:** ^1^Department of Cerebrovascular Diseases, The Second Affiliated Hospital of Zhengzhou University, Zhengzhou, China; ^2^Academy of Medical Science, Zhengzhou University, Zhengzhou, China; ^3^Department of Clinical Neurosciences, Hotchkiss Brain Institute, University of Calgary, Calgary, AB, Canada

**Keywords:** intracerebral hemorrhage, necrosulfonamide, mixed lineage kinase domain-like protein, necroptosis, neuroinflammation, blood-brain barrier

## Abstract

**Objective:**

Intracerebral hemorrhage (ICH) is the most lethal subtype of stroke, without effective treatment. Necrosulfonamide (NSA), a specific inhibitor for mixed lineage kinase domain-like protein, has been reported to exert neuroprotective effects in neurological diseases by ameliorating neuroinflammation and necroptosis. We hypothesized that NSA would alleviate acute brain injury and improve behavioral outcomes after ICH.

**Materials and Methods:**

Male adult C57BL/6 mice were assigned randomly into three groups. In vehicle and treatment groups, animals were injected with collagenase VII to induce ICH. The solvent (0.25% DMSO) and NSA (5 mg/kg) were administrated intraperitoneally twice a day, respectively. The sham group was injected with saline and administrated with DMSO. The brain hematoma volume, inflammatory factors, and blood-brain barrier permeability were measured on day 3 after the operation. Fluorescent double immunostaining was performed to evaluate the neuronal death. Neurological functions were assessed.

**Results:**

In the NSA group, the hematoma size was significantly reduced, inflammatory cells and cytokines were suppressed, and the blood-brain barrier was protected compared to vehicle controls. NSA dramatically reduced the death of neurons and improved the performance of neurological functions after ICH.

**Conclusion:**

Necrosulfonamide has a neuroprotective role in alleviating acute brain injury in a mouse ICH model, and this is associated with reduced neuroinflammation and necroptosis.

## Introduction

Intracerebral hemorrhage (ICH) is defined as a primary, spontaneous, and non-traumatic hemorrhage of brain parenchyma, and represents the most fatal type of stroke although it constitutes 15–20% of strokes (Samarasekera et al., 2015; Xue and Yong, 2020). The mortality rate within 1 year after ICH is approximately 40% (Sacco et al., 2009), and only 39% of survivors showed functional independence (Asch et al., 2010). The poor prognosis is caused by the primary injury induced by extravasated blood along with soaring intracranial pressure, which compromises cerebral blood flow; secondary injuries of inflammation, cell death, and destruction of the blood–brain barrier (BBB) also contribute to the poor prognosis (Keep et al., 2012). The early and precise treatment against secondary brain injury is critical for a patient’s prognosis after ICH. However, there has been no effective therapy up to now.

Among various pathways of cell death, necroptosis has gained prominence in recent years. Necroptosis, which can be triggered by tumor necrosis factor (TNF) receptor and Toll-like receptor (TLR) activation, is increasingly considered one of the most crucial processes contributing to secondary injury after ICH (Liesz et al., 2011; Wang et al., 2014c). It relies on intracellular signals, consisting of receptor-interacting protein kinase-1 (RIPK1), receptor-interacting protein kinase-3 (RIPK3), and mixed lineage kinase domain-like protein (MLKL) (Degterev et al., 2005). The membrane translocation of MLKL oligomers disrupts cellular integrity and increases the permeability of the membrane, therefore, inducing the inward flow of Ca^2+^ or Na^+^, leading to cell death (Wang et al., 2014b). MLKL is a key molecule mediating the execution of necroptosis and might be a more specific target for inhibition of necroptosis than other downstream components (Sun et al., 2012; Zhao et al., 2012).

A series of agents aimed at ameliorating necroptosis have improved neurological results in ICH (Shen et al., 2017; Yang et al., 2021). Necrosulfonamide (NSA) is a specific inhibitor of MLKL. Studies have shown neuroprotective effects of NSA in spinal cord injury and other neurological diseases (Jiao et al., 2019; Motawi et al., 2020; Zhang et al., 2020). Whether NSA has a protective role in ICH is undetermined. We tested the hypothesis that NSA would alleviate acute brain injury after ICH and improve neurological recovery. To address this hypothesis, we investigated inflammation, neuronal death, BBB permeability, and neurological function after ICH by using hematoma volume measurement, immunostaining, western blot, Evans blue (EB) staining, and behavioral functions.

## Materials and Methods

### Establishment of the Intracerebral Hemorrhage Model and Experimental Groups

In this study, 108 healthy adult male C57BL/6 mice (20–25 g, 8–10 weeks) were used, provided by Beijing Vital River Experimental Animals Centre (Beijing, China). All animals were housed in a Specified Pathogen Free room with a temperature set at 25°C, under 12 h of light/dark circulation, and they had free access to food and water. Our experimental protocols were approved by the Committee on the Use and Care of Animals of Zhengzhou University. In addition, all animal experiments were approved by the Ethics Committee of Zhengzhou University, according to the China Council on Animal Care guidelines. Mice were randomly divided into three groups: sham group, vehicle (ICH + DMSO) group, and treatment (ICH + NSA) group, marked with particular ear tag numbers.

We established the ICH model based on previous literature with slight modifications (Rosenberg et al., 1990). We first anesthetized mice with 4% chloral hydrate (400 mg/kg). Next, a diameter of 0.6 mm burr hole was made on top of the following coordinates: 0.8 mm anterior to the bregma and 1.5 mm lateral to the midline. Then, we slowly injected 0.075 U (0.1 U/μl) collagenase VII (Sigma, United States) at a rate of 0.1 μl/min (Zhang et al., 2022b) into the basal ganglia area at a depth of 3.5 mm, with syringe remaining for another 10 min to prevent backflow. The counterparts in the sham group were injected with 0.75 μl saline instead. The animal procedure was performed by skilled study personnel to minimize any discomfort and stress during the experiment. After the operation, food was placed on the cage floor, and mice were kept warm until recovered completely. Food and water intake, body weight, and general behavior of the mice were monitored daily.

### Drug Treatment

After collagenase injection, the mice in the treatment group were administrated NSA (5 mg/kg, Selleck, Shanghai, China), first, it was dissolved in dimethyl sulfoxide (DMSO), and then diluted in saline. Their counterparts in the vehicle group were treated with an equivalent amount of 0.25% DMSO 2 times a day intraperitoneally. The dose of NSA was based on an earlier study (Jiao et al., 2019). For sham-operated mice, we gave the same dose of DMSO for treatment.

### Histological Processing

#### Tissue Preparation

Normally, microglia reaction and neutrophil infiltration in the brain adjacent to the hematoma is obvious at 2–3 days after ICH (Bai et al., 2020), and BBB disruption is most severe 3 days after ICH (Liu et al., 2021). Therefore, brain specimens were collected 72 h after the operation, after a deep anesthetic using an overdose of chloral hydrate. In brief, mice were transcardially perfused with ice cold saline and paraformaldehyde (Servicebio, Wuhan, China), then the brains were removed and fixed for 24 h to 3 days in tissue fixation fluid, and were dehydrated and paraffin-embedded for *post-hoc* histological analysis. We harvested and trimmed the brain tissues 3 mm around the hematoma. After that, they were cut into a set of 5-μm thick coronal sections for staining. For protein analysis, the fresh brain tissues were harvested from 1 to 1.5 mm around the hematoma.

#### Hematoma Volume

The fresh brain specimens were coronally sectioned, each 1 mm thick. The total ICH hematoma volume was calculated by summing the hemorrhagic area, using Coniglobus Formula, as has been used in other studies (Wang et al., 2014a). The volume was based on this formula 1/2 × the longest diameter of the hematoma layer with the largest area on axial cuts (cm) × the diameter perpendicular to the longest diameter mentioned (cm) × the thickness of the hematoma (cm). Open-source image processing software Image J/Fiji was used to measure the volume.

#### Immunofluorescence Staining

After dewaxing the paraffin sections of brain tissue with dimethylbenzene and graded ethanol, antigen retrieval was performed by incubating the sections in a retrieval buffer at 100°C for 3 min and 40°C for another 15 min. Sections were then blocked in 5% bovine serum albumin for 1 h at room temperature. Subsequently, all sections were incubated overnight with a rabbit anti-Iba1 polyclonal antibody (microglia marker, 1:300, Wako, Japan) at 4°C. After rinsing with PBS (pH = 7.4) thrice, the corresponding secondary antibody (the Alexa Fluor 488-conjugated goat anti-rabbit) (1:500, Abcam, Cambridge, MA, United States) was added for 1 h at room temperature. All sections were cover slipped using an anti-fade mounting medium, and then were observed under a fluorescent microscope (Olympus Co., Tokyo, Japan). Immunoreactivity was examined at optimal resolution. Resting microglia are recognized as highly branched ramified cells with small cell bodies, and activated Iba-1cells are ameboid, rounded cells with few processes (Xue et al., 2010). Four microscopic fields in each brain section and three sections per tissue were detected for positive cells. Under uniform shooting conditions, the number of Iba1-positive cells was counted by Image J/Fiji.

#### Immunohistochemical Staining

The preceding few steps were identical to those used for tissue immunofluorescence (IF). But before blocking, brain sections (*n* = 6 per group) were incubated for 15 min at room temperature into a mixed solution (3% H_2_O_2_). After incubation with rabbit anti-myeloperoxidase (MPO) monoclonal antibody (1:500, Abcam, Cambridge, MA, United States) at 4°C overnight, the sections were washed three times in PBS before adding the horseradish peroxidase (HRP)-combined secondary anti-rabbit immunoglobulin G (IgG) antibody (1:800, Servicebio, Wuhan, China) and incubating for 1 h at room temperature on an orbital shaker. Then all sections were exposed to diaminobenzidine (DAB) reagent for 30 min, followed by hematoxylin. After dehydration, the sections were protected with coverslips.

#### Western Blot Analysis

Brain tissue from perihematomal region was lysed in ice-cold Radio-Immunoprecipitation Assay (RIPA) buffer (Solarbio, Beijing, China) supplemented with a protease inhibitor cocktail (Beyotime, Nanjing, China). Then, 20 μg of protein per sample were resolved on sodium dodecyl sulfate-polyacrylamide gel electrophoresis (SDS-PAGE) and transferred onto polyvinylidene difluoride (PVDF) membranes. The membranes were incubated for 1 h at room temperature in tris-buffered saline with Tween 20 (TBST) containing BSA and then incubated with primary antibodies including rabbit anti-MMP-9 polyclonal antibodies (1:1,000, Abcam, Cambridge, MA, United States), rat monoclonal [3H1] to MLKL (1:500, Abcam, Cambridge, MA, United States), rabbit RIPK1-specific polyclonal antibody (1:500, ProteinTech Group, Chicago, IL, United States), rabbit RIP3 polyclonal antibody (1:500, ProteinTech Group, Chicago, IL, United States), rabbit anti-ZO-1 monoclonal antibody (1:5,000, Abcam, Cambridge, MA, United States), and rabbit anti-GAPDH monoclonal antibody (1:2,000, Servicebio, China) overnight at 4°C. The blots were then washed three times in TBST and incubated in secondary antibodies including HRP-conjugated anti-rabbit IgG antibody (Servicebio, Wuhan, China) and HRP-conjugated goat anti-rat IgG (H + L) antibody (Servicebio, Wuhan, China) at room temperature for 1 h. The protein bands were detected using Amersham Imager 600 (GE, United States). Optical density (OD) of signals was analyzed by Image J/Fiji.

#### Terminal Deoxynucleotidyl Transferase-dUTP Nick-End Labeling and Immunofluorescent Staining

Immunofluorescence double staining of terminal deoxynucleotidyl transferase-dUTP nick-end labeling (TUNEL) (Vazyme Biotech, Nanjing, China) and NeuN (1:100, Abcam, Cambridge, MA, United States) were conducted to determine neuronal death. After double labeling with NeuN, and TUNEL staining, DAPI (4′, 6-diamidino-2-phenylindole) was used to stain the nuclei. Negative control was similarly performed except for omitting the TUNEL reaction mixture. Immunofluorescencence was visualized on a fluorescence microscope (Olympus Co., Tokyo, Japan) by an investigator who was blinded to the experiment, and four randomly chosen high powered fields per section were analyzed. Dead neurons were those positive cells combined into white color. The counting was performed using the Image J/Fiji software.

#### Evans Blue Staining

Blood-brain barrier permeability was assessed at 72 h after ICH. EB dye (2%, 5 ml/kg) was injected over 2 min into the caudal vein and allowed to circulate for 4 h. The amount of extravasated Evans blue dye in the brain was determined by spectrofluorophotometry. Measurements were conducted at an excitation wavelength of 630 nm.

### Neurological Function Assessments

We conducted behavior tests to evaluate the neurological function of ICH mice.

#### Corner Test

All mice were taken to a 30-degree corner, formed by two 20-cm long boards. Each mouse was placed between the two boards, half way to the corner. When mice entered deep into the corner, we recorded the left or right direction they chose to turn. The test was repeated 20 times for each animal, and the percentage of right turns was counted.

#### Focal Deficits Neurological Scores

A 28-point neurologic deficit scale was used to assess neurologic deficits, including body symmetry, gait, climbing, circling behavior, front limb symmetry, compulsory circling, and whisker response, as previously mentioned (Fang et al., 2014; Xu et al., 2020). A higher score represents a more severe injury. Mice were tested for three consecutive days before operation to exclude abnormal mice and this form baseline data. Then neurologic deficit scale was measured on days 1 and 3 after ICH. The scoring was performed in a blinded way by two experienced investigators.

### Statistical Analysis

Data were presented as the mean ± standard deviation (SD). Comparisons between three independent groups were performed using the ANOVA along with Bonferroni tests. All statistical analyses were performed with GraphPad Prism. Differences at *p* < 0.05 were considered significant.

## Results

### Necrosulfonamide Reduced the Size of the Hematoma

**FIGURE 1:**
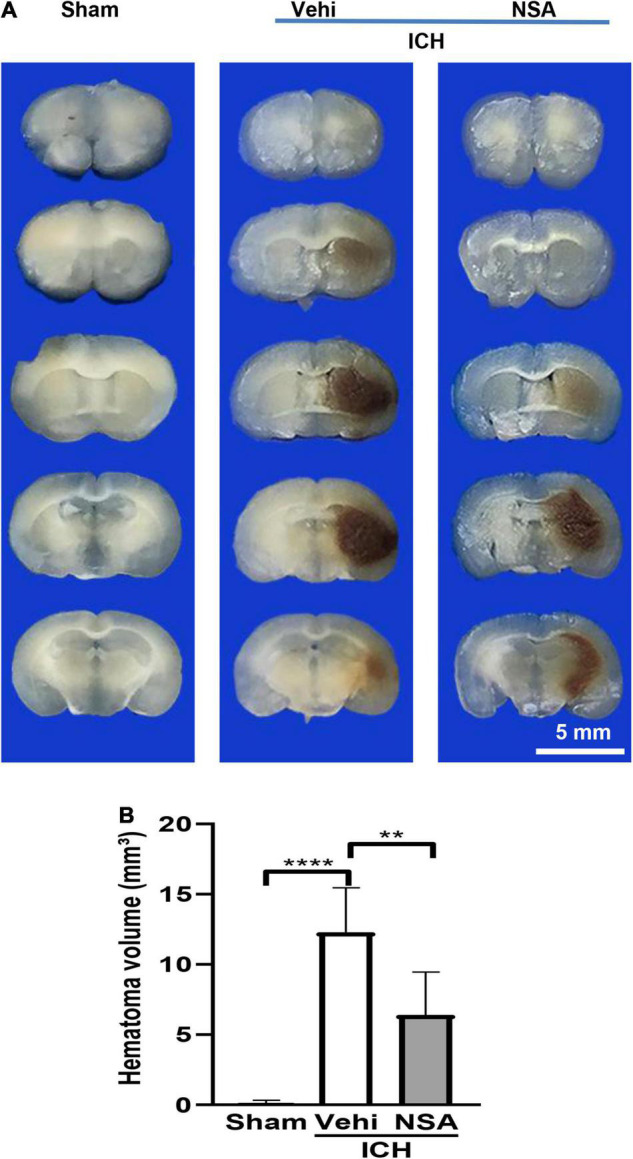
Necrosulfonamide (NSA) reduced the hematoma volume of intracerebral hemorrhage (ICH). **(A)** Representative images of hematoma volume after ICH. **(B)** Quantification analysis indicates that NSA significantly reduced hematoma volume at 3 days after ICH. Data are shown as mean ± SD (*n* = 6, per group); scale bar = 5 mm; **p < 0.01, ****p < 0.0001.

Hematoma volume was calculated as a measure of brain injury after ICH. We analysed six mouse brains per group. As shown in Figure 1, there was a smaller size of hematoma around the injury area after the NSA treatment compared with the vehicle group. No evidence of obvious brain damage was observed in the sham group injected saline intracerebrally. These results show that NSA treatment significantly reduced brain damage in the ICH mouse model.

### Necrosulfonamide Suppressed Inflammation

**FIGURE 2:**
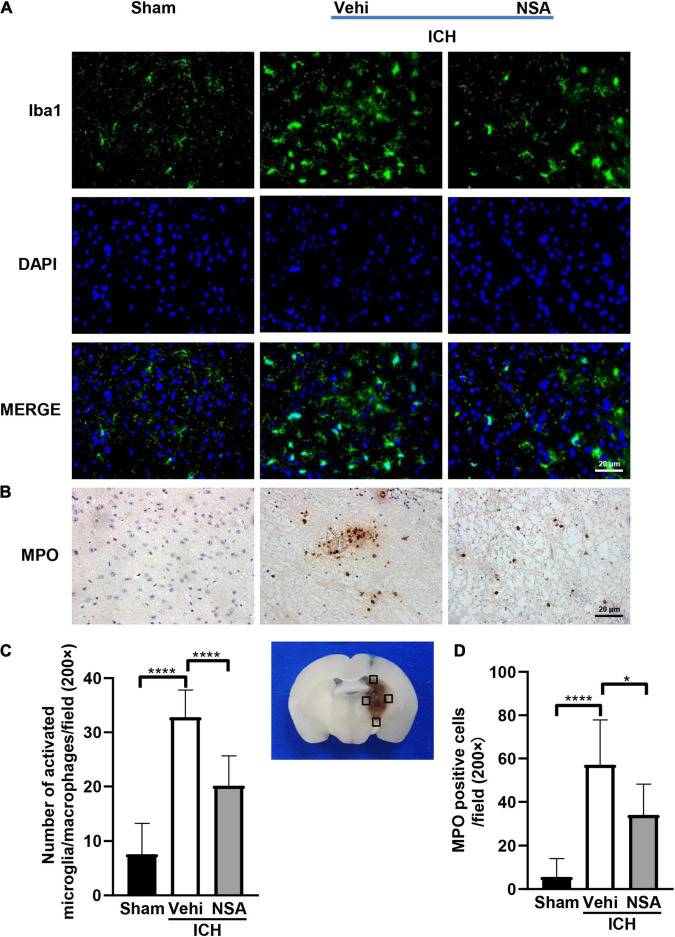
Necrosulfonamide (NSA)repressed the activation of microglia/macrophages and neutrophil infiltration at 3 days after intracerebral hemorrhage (ICH). **(A)** Representative photographs of Iba1 expression in the perihematomal area at 3 days after ICH. **(B)** Representative photographs of immunohistochemical staining for neutrophil infiltration in the perihematomal area at 3 days after ICH. **(C)** Quantification shows that NSA ameliorated the activation of microglia/macrophage. **(D)** NSA inhibited the infiltration of neutrophils. Data are shown as mean ± SD (*n* = 6, per group); scale bar = 20 μm; *p < 0.05, ****p < 0.0001.

To assess the effects of NSA on inflammatory reactions, six mouse brain tissues per group were analyzed at 3 days post-ICH. We evaluated neuroinflammation after ICH by immunofluorescent and histochemical staining. Figure 2 shows representative images of Iba1 and MPO positive cells. Activated microglia/macrophages were significantly reduced by NSA, which also caused a smaller quantity of infiltrated neutrophils around the lesion, compared to the ICH control group. Collectively, these data show that NSA significantly reduced neuroinflammation at day 3 post-ICH.

### Necrosulfonamide Inhibited Necroptosis Following Intracerebral Hemorrhage in Mice

**FIGURE 3:**
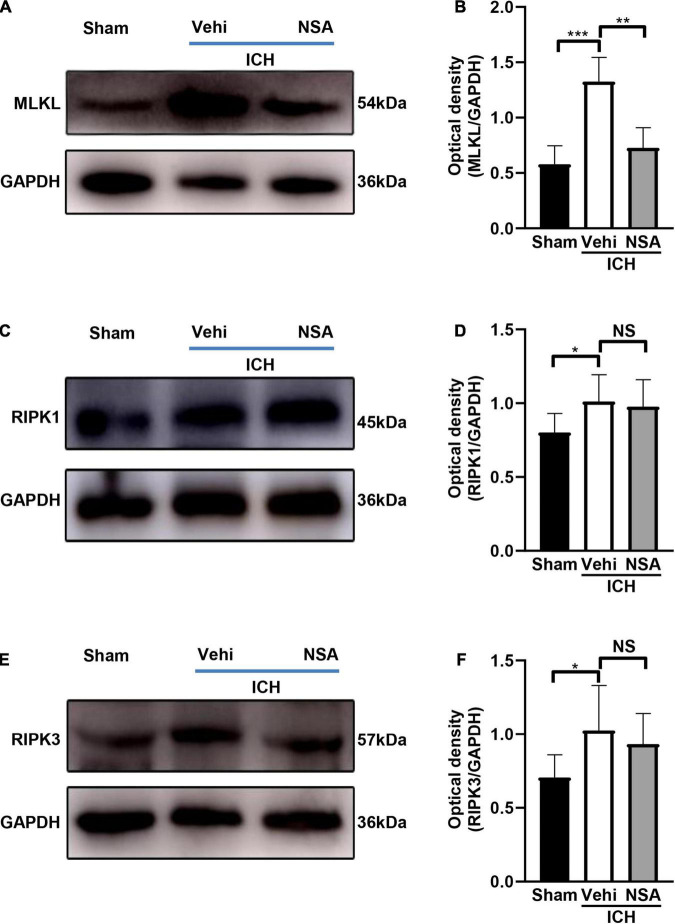
The effects of necrosulfonamide (NSA) on the expression of MLKL, RIPK1, and RIPK3 at 3 days after intracerebral hemorrhage (ICH). **(A,C,E)** Representative western blotting images of the expression of MLKL, RIPK1, and RIPK3, respectively. **(B,D,F)** Quantification shows that NSA reduced the expression of MLKL (*n* = 3, per group), but not of RIPK1 (*n* = 3, per group) or RIPK3 (*n* = 3, per group) after ICH. Data are shown as mean ± SD, *p < 0.05, **p < 0.01, ***p < 0.001.

**FIGURE 4:**
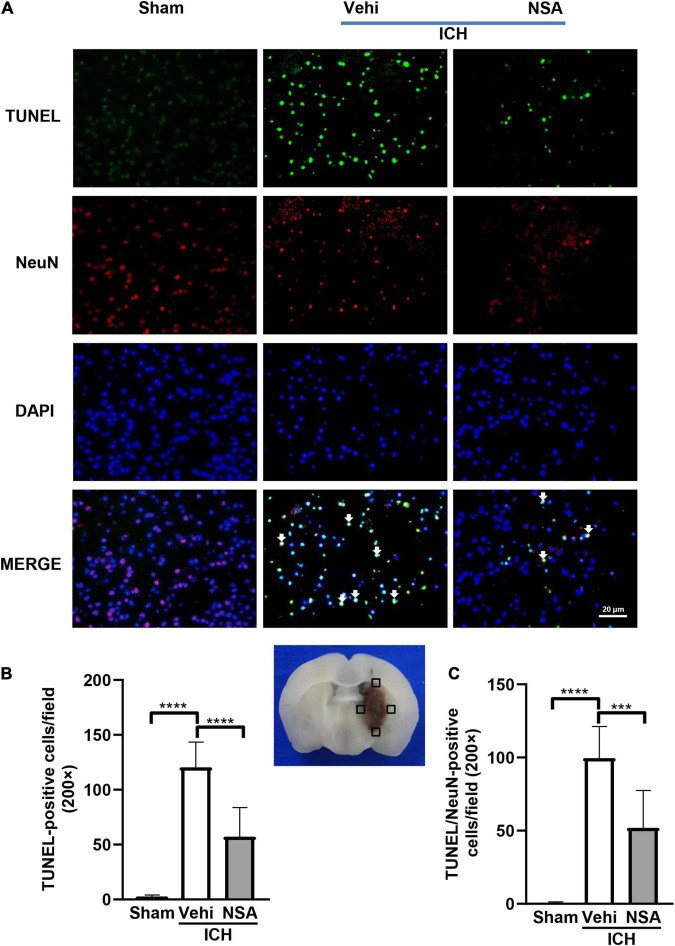
The effect of necrosulfonamide (NSA) on death of neurons. **(A)** Representative photographs of TUNEL positive cells, NeuN positive cells, and TUNEL and NeuN double positive cells (white arrows) in the perihematomal area. **(B)** Quantitative analysis of TUNEL positive cells in the perihematomal area at 3 days after intracerebral hemorrhage (ICH). **(C)** Quantitative analysis of TUNEL positive neurons in the perihematomal area at 3 days after ICH. Data are shown as mean ± SD (*n* = 6, per group); scale bar = 20 μm; ***p < 0.001, ****p < 0.0001.

Mixed lineage kinase domain-like protein, RIPK1, and RIPK3 are pivotal indicators of necroptosis. TUNEL staining is an effective method to detect DNA breakage by labeling the free 3’-hydroxyl termini, which are generated not only in apoptotic cells but also in other modes of death including necroptosis (Ueda et al., 2021). Six mice per group were included for TUNEL staining, and three brains per group were used to assess the expression level of proteins. Western blot analyses showed that MLKL level in the NSA group was significantly decreased compared with vehicle controls (Figures 3A,B). As to RIPK1 and RIPK3, another two components that may affect cell death, NSA administration did not show a significant impact on their expression levels (Figures 3C–F). TUNEL staining to evaluate cell death in NeuN-positive neurons found that dead cells (positive only for TUNEL) were stained green, neurons were stained red, and dead neurons were stained white when DAPI was added (Figure 4A). NSA administration significantly reduced the mean number of dead cells (Figure 4B) and dead neurons (Figure 4C) compared to the vehicle group. Thus, NSA administration reduce neuronal death after ICH, possibly by inhibiting necroptosis.

### Necrosulfonamide Protected the Blood-Brain Barrier After Intracerebral Hemorrhage

**FIGURE 5:**
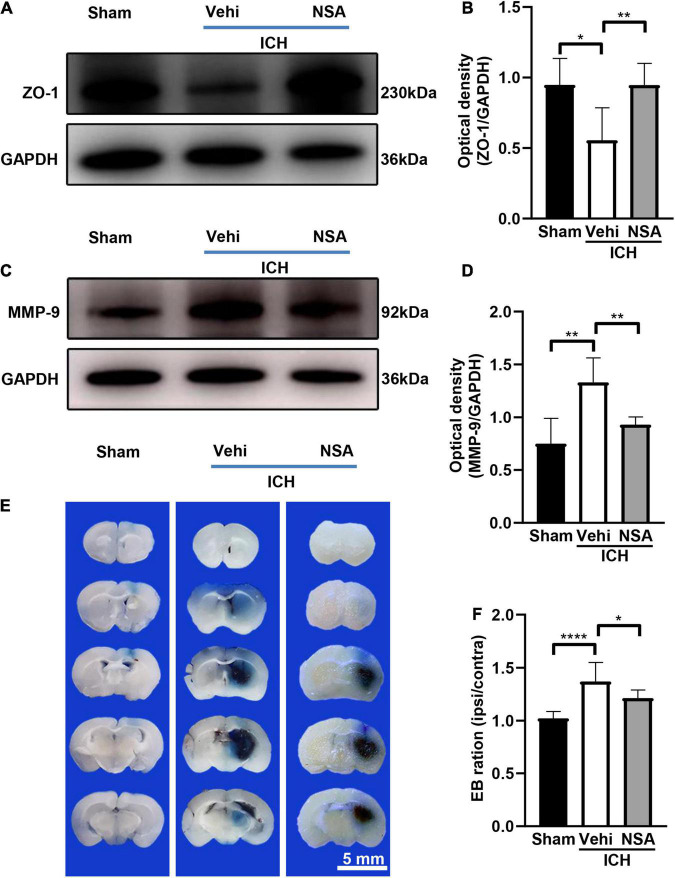
The effect of necrosulfonamide (NSA) on blood-brain barrier permeability in mice after intracerebral hemorrhage (ICH). **(A)** Representative western blotting images of the expression of ZO-1 at 3 days after ICH. **(B)** Quantification shows that NSA increased the expression of ZO-1 (*n* = 3, per group). **(C)** Representative western blot images of the expression of MMP-9 at 3 days after ICH. **(D)** Results show that NSA decreased the expression of MMP-9 (*n* = 3, per group). **(E)** Representative images of Evans blue dye leakage at 3 days after ICH. **(F)** Evans blue leakage at 3 days after ICH (*n* = 10, per group). Data are shown as mean ± SD; scale bar = 5 mm; *p < 0.05, **p < 0.01, ****p < 0.0001.

Western blot and EB staining were used to assess the damage to the blood-brain barrier. We conducted 10 independent experiments for EB staining. Figure 5 shows representative images of ZO-1 and MMP-9 expression, and EB staining at 3 days after collagenase or saline injection in mice. As found by western blots, the expression of ZO-1 in NSA group was higher compared to that in the vehicle group. The increased level of MMP-9 after ICH in the vehicle group was reduced by NSA. As determined by EB leakage, there was less coloring agent permeating across the blood-brain barrier into the parenchyma after NSA treatment. These results indicate that BBB was broken after ICH and that the administration of NSA significantly reduce permeability and protected the BBB.

### Necrosulfonamide Improved Neurological Function

**FIGURE 6:**
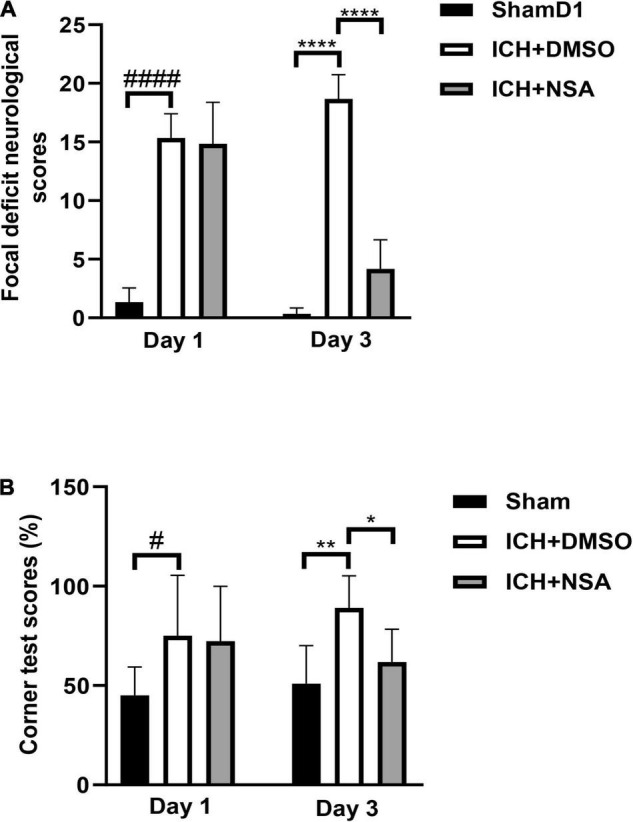
Necrosulfonamide (NSA) improved neurological functions after intracerebral hemorrhage (ICH). **(A)** Quantification of focal neurological deficit at 3 days after ICH (*n* = 6, per group). **(B)** Quantification of corner test scores at 1 day and 3 days after ICH (*n* = 11, per group). Data are shown as mean ± SD, *p < 0.05, **p < 0.01, ****p < 0.0001, ^#^*p* < 0.05, ^####^*p* < 0.0001.

To determine whether NSA improved motor and sensory functions after ICH, we subjected mice to a battery of behavior tests at 24 and 72 h after injury. Focal deficit neurological scores and corner tests were evaluated 6 and 11 times, separately. All ICH groups had the same baseline pre-ICH, and after ICH at 24 h (Figure 6A), the vehicle and NSA groups displayed similar neurological impairments, with marked focal contralateral motor deficits. At 3 days, following ICH, however, focal deficit neurological scores were lower in the NSA compared to the vehicle group.

In the corner test, the right-turn rate of normal mice was approximately 50%. At 1 day after ICH, the rate of turning for both the vehicle and treatment group was similar. However, at 3 days, after ICH, the percentage of right turn of the NSA group trended toward a significantly lower extent compared to the vehicle group (Figure 6B).

## Discussion

In this study, we discovered that the administration of NSA significantly alleviated acute brain injury after ICH. Our data showed that the volume of hematoma was significantly decreased after NSA intervention in the ICH mice model (Figure 1). NSA administration reduced the representation of activated microglia/macrophages and neutrophils (Figure 2) and lowered the expression of MMP-9 (Figure 5). Western blot analysis found the reduction of MLKL by NSA while having no effect on RIPK1 and RIPK3 (Figure 3). In addition, fluorescent double immunostaining found a lower number of dead neurons following ICH in NSA-treated animals (Figure 4), and protection of the BBB (Figure 5). These changes were associated with a better neurological function score, as indicated by focal deficit assessment and corner test, after NSA administration (Figure 6).

Necrosulfonamide is known to prevent necroptosis via specifically suppressing MLKL, a key mediator for the early destruction of membrane integrity in the necroptotic pathway (Zhang et al., 2016). Previous reports have shown that NSA was able to improve spinal cord injury (Jiao et al., 2019), or alleviated rat pulmonary ischemia-reperfusion injury via the attenuation of inflammation and inhibition of necroptosis (Dong et al., 2017). However, as far as we know, this is the first research exploring the potential neuroprotective effect of NSA in ICH. The results support the view that NSA can effectively alleviate acute brain injury of ICH, as this is due possibly to repressing inflammation and necroptosis.

Inflammation essentially manifests immediately after ICH and persists for several days, which may exacerbate the progress of secondary brain injury (Wu et al., 2010). Microglia/macrophages, as one kind of inflammatory participant, become activated and phagocytic within a few minutes of the initial bleeding (Lan et al., 2017). In correspondence, the activation of the peripheral immune system allows leukocytes to migrate via the broken BBB into the damaged site (Mracsko and Veltkamp, 2014). In the initiation and development of inflammatory reactions, these immune cells play pivotal roles. Several pro-inflammatory mediators are elevated after ICH (Zhou et al., 2014; Zhu et al., 2019). But after NSA injection, MMP-9 was decreased in the NSA group. Our data suggest that NSA can effectively repress neuroinflammation after ICH.

Neurons are essential for the ability of think and move. We applied the double fluorescent staining method of TUNEL, which is sensitive to detecting dead cells, and a neuronal-specific nuclear protein NeuN to observe the neuronal death in this study. The data illustrated that NSA distinctly reduced ICH-associated neuronal death. RIPK1, RIPK3, and MLKL form the core components of necroptosis and MLKL is the substrate of the other two (Degterev et al., 2005). NSA is a specific inhibitor of MLKL while it does not appear to directly act on RIPK1 and RIPK3. In support, we observed that NSA lowered MLKL but not RIPK1 and RIPK3 in the ICH homogenates. These findings suggest that NSA inhibits the death of neurons after ICH by suppressing necroptosis. Follow-up research concerning phosphorylated MLKL will be further studied to support our work.

Based on previous studies, necroptosis can be activated by several events, such as TNF release, TLR activation, and inflammation, which occur in the brain following ICH, and activated microglia as a prominent factor leading to necroptosis (Liesz et al., 2011; Wang et al., 2014c; Zhang et al., 2022a). Necroptosis can promote inflammation by inducing the production of inflammatory cytokines, and it has roles in the development of diseases such as multiple sclerosis, myocardial ischemia-reperfusion injury, and dermatitis (Linkermann and Green, 2014). Necroptosis can elicit a strong inflammatory response that can drastically alter the local tissue environment (Christofferson et al., 2012). The proinflammatory effect of necroptosis is not only due to cell disintegration and the release of damage associated molecular patterns (DAMPs), but also the involvement of RIPK1, RIPK3, and MLKL in the activation of inflammasomes, which are responsible for the secretion of IL-1β and IL-18 (Kang et al., 2015). These may contribute to NSA inhibiting necroptosis along with suppressing inflammation after ICH.

After ICH, BBB damage can result from pro-inflammatory cytokines and reactive oxygen species, which are secreted by the excess activation of microglia (Taylor et al., 2017). As well, the MMP-9 produced after ICH can not only enhance inflammation, and perihematomal edema, and cause lesional expansion, but also contribute to BBB disruption by degrading the extracellular matrix (Zhang et al., 2021). Therefore, the NSA-reduced MMP-9 (Figure 5) may help protect the BBB. It could be clearly noticed that ZO-1, which maintains the integrity of BBB, expressed a lot more in the NSA group than that in the vehicle group in our experiments. Furthermore, the results of EB extravasation indicated that the BBB in the treatment group mice functioned better than their counterparts in the vehicle group.

Neurological deficits occur after ICH. In our model, the animals were scored at 3 days using a 28-point neurological scores assessment and corner tests. We found that NSA improved recovery after ICH compared to the vehicle group. The volume of hematoma is a key factor that affects the prognosis of patients with ICH (Davis et al., 2007). We found that the volume of hematoma was significantly lower after NSA treatment. These data show that the administration of NSA ameliorates behavioral and pathological outcomes of ICH mice.

Several limitations of this study should be noted. In our study, the necroptosis inhibitor was administered immediately after ICH; while significant improvements were seen in the NSA group, more detailed data including a delayed treatment should be obtained in the future. Another limitation is the 72 h analysis after ICH, and longer-term studies should be considered. Regarding safety, although no obvious toxicity from NSA administration was detected in rat liver, kidney, heart, and spleen (Wang et al., 2018), potential side effects of concern remain to be verified in future studies. In addition, previous studies have shown that estrogen can play a neuroprotective role in models for acute neuronal injury and death (Simpkins et al., 2012), which may affect the results; therefore our experiments only used male animals. It is still unclear the effects of NSA on females.

## Conclusion

In conclusion, we have demonstrated that NSA, an inhibitor of MLKL, attenuates ICH associated with inhibition of inflammation and suppression of necroptosis. The NSA intervention significantly improved neurological functions and reduced neuropathology after ICH. The inhibition of necroptosis by an inhibitor such as NSA appears to be a promising new option for ICH treatment.

## Data Availability Statement

The original contributions presented in this study are included in the article/supplementary material, further inquiries can be directed to the corresponding authors.

## Ethics Statement

The animal study was reviewed and approved by the Ethics Committee of Zhengzhou University.

## Author Contributions

XZ and MX designed the experiments. XZ wrote the manuscript. XZ, YZ, FW, and YL collected, analyzed, and interpreted the data. VY consulted and edited the manuscript. MX supervised the project. All authors discussed the results and agreed on the content of the manuscript.

## Conflict of Interest

The authors declare that the research was conducted in the absence of any commercial or financial relationships that could be construed as a potential conflict of interest.

## Publisher’s Note

All claims expressed in this article are solely those of the authors and do not necessarily represent those of their affiliated organizations, or those of the publisher, the editors and the reviewers. Any product that may be evaluated in this article, or claim that may be made by its manufacturer, is not guaranteed or endorsed by the publisher.

## Abbreviations

BBB: blood-brain barrier
DAB: diaminobenzidine
DMSO: dimethyl sulfoxide
EB: Evans blue
HRP: horseradish peroxidase
ICH: intracerebral hemorrhage
NSA: necrosulfonamide
MLKL: mixed lineage kinase domain-like protein
MPO: myeloperoxidase
RIPK1: receptor-interacting protein kinase-1
RIPK3: receptor-interacting protein kinase-3
TNF: tumor necrosis factor
TLR: toll-like receptor
TUNEL: terminal deoxynucleotidyl transferase-dUTP nick-end labeling.
